# Mechanisms governing the reactivation-dependent destabilization of memories and their role in extinction

**DOI:** 10.3389/fnbeh.2013.00214

**Published:** 2013-12-26

**Authors:** Charlotte R. Flavell, Elliot A. Lambert, Boyer D. Winters, Timothy W. Bredy

**Affiliations:** ^1^Queensland Brain Institute, The University of QueenslandBrisbane, QLD, Australia; ^2^Department of Psychology, University of GuelphGuelph, ON, Canada

**Keywords:** extinction, reconsolidation, destabilization, reactivation, memory, fear

## Abstract

The extinction of learned associations has traditionally been considered to involve new learning, which competes with the original memory for control over behavior. However, a recent resurgence of interest in reactivation-dependent amnesia has revealed that the retrieval of fear-related memory (with what is essentially a brief extinction session) can result in its destabilization. This review discusses some of the cellular and molecular mechanisms that are involved in the destabilization of a memory following its reactivation and/or extinction, and investigates the evidence that extinction may involve both new learning as well as a partial destabilization-induced erasure of the original memory trace.

## Introduction

Under certain conditions, memories can be rendered temporarily labile and sensitive to modification, after which they must be re-stabilized through a process called reconsolidation (Lewis et al., 1972; Przybyslawski et al., 1999; Nader et al., 2000; Lee, 2009; Finnie and Nader, 2012). Once memories are destabilized it is also possible to enhance (Tronson et al., 2006; Lee, 2008; Debiec et al., 2011; Tian et al., 2011) and even incorporate new information into existing memories (Winters et al., 2009; Choi et al., 2010; Lee, 2010; Winters et al., 2011), which has led to the suggestion that reactivation-induced destabilization of memory is an important updating mechanism that is required for new learning (Lee, 2009). In models of associative learning, destabilization of previously acquired memory traces can be achieved by re-exposure to a conditioned stimulus (CS; e.g., tone) in the absence of the unconditioned stimulus (US; e.g., a foot shock). This can promote either reconsolidation of the original memory or extinction depending on several boundary conditions, including stimulus intensity, training to test interval, or the duration of the reminder cue (Eisenberg et al., 2003; Pedreira and Maldonado, 2003; Pedreira et al., 2004; Suzuki et al., 2004; Lee et al., 2006; Power et al., 2006; Tronson et al., 2006; Nader and Hardt, 2009; Wang et al., 2009; Reichelt and Lee, 2012; Flavell and Lee, 2013). If reconsolidation is interrupted, memories may also be prevented from returning to a stable state, which can lead to amnesia (Lewis et al., 1972; Przybyslawski and Sara, 1997; Nader et al., 2000; Lee et al., 2006). Conversely, if processes related to extinction are interrupted, the relative strength of the memory will remain unchanged and presentation of the CS will continue to elicit conditioned behavior (Eisenberg et al., 2003; Pedreira et al., 2004; Suzuki et al., 2004; Flavell and Lee, 2013).

Retrieval refers to a process whereby the activation of neuronal networks leads to the recall of a memory, allowing the expression of an appropriate behavioral response. However, this does not necessarily imply that when a memory is retrieved it is also reactivated. Retrieval and reactivation are independent processes and there are accounts of memories being retrieved but not susceptible to change (Cammarota et al., 2004). Reconsolidation refers to the molecular mechanisms required to return a memory to a stable state, therefore, for a memory to be reconsolidated it must have first entered a reactivated state and become destabilized.

Extinction training is commonly used to reduce aberrant emotional responses associated with phobias or post-traumatic stress disorder (PTSD; Rauch et al., 2012) and results in a reduction in the strength of a given memory, due to repeated exposure to the CS in the absence of the US. The general consensus is that extinction does not affect the original memory trace as it is prone to spontaneous recovery (return of fear over time), reinstatement (return of fear following unsignaled presentation of the US) and renewal (return of fear following the presentation of contextual cues) (Myers and Davis, 2007). However, a reconsolidation-extinction paradigm has recently been introduced, whereby extinction training applied within the reconsolidation window, i.e., when the memory is in a destabilized state, leads to a permanent reduction in the expression of fear behavior. This reduction is resistant to renewal, reinstatement and spontaneous recovery (Monfils et al., 2009; Schiller et al., 2010; but see Chan et al., 2010; Kindt and Soeter, 2013), indicating that the original memory trace can indeed be modified. A reactivation session and an extinction session differ only in terms of their duration and/or frequency, yet they can lead to very different outcomes. Reactivation followed by reconsolidation can serve to strengthen or update a memory, extinction leads to the formation of a new competing memory, and a reactivation-extinction session appears to permanently affect a previously stable memory trace. Therefore, it is likely that the molecular mechanisms invoked at the time of reactivation are critical for determining the consequences of what are procedurally very similar behavioral manipulations. An understanding of these mechanisms is likely to have great therapeutic relevance for the treatment of PTSD, phobia and addiction. The purpose of this review is to discuss some of the cellular and molecular mechanisms involved when a memory is retrieved and subsequently extinguished, and what impact this process is likely to have on the original memory trace.

## Dendritic spine remodeling

In its simplest form, memory can be viewed as the strengthening of synaptic connections, which occurs through the experience-dependent structural remodeling of dendritic spines. Dendritic spine formation and elimination have both been associated with the formation of new memories (for review see Yuste and Bonhoeffer, 2001; Lamprecht and Ledoux, 2004; Bailey and Kandel, 2008; Caroni et al., 2012). Structural modification to dendritic spines occurs *in vivo* in both invertebrates (Bailey and Chen, 1983, 1988a,b,1989a,b) and mammals (Geinisman et al., 2001; Knafo et al., 2001; Kleim et al., 2002; Leuner et al., 2003; Restivo et al., 2009; Xu et al., 2009; Yang et al., 2009; Fu et al., 2012; Lai et al., 2012).

In an emotional learning paradigm, the changes in dendritic spines that occurr following fear acquisition are opposed to those that occur after extinction, For example, fear conditioning induces spine elimination within the prefrontal cortex (PFC), whereas fear extinction increases the rate of spine generation (Vetere et al., 2011; Lai et al., 2012). Additionally, it has been demonstrated that spine formation induced by extinction occurs within very close proximity to the original position of spines that were previously eliminated by acquisition, thereby illustrating that increases in spine density following extinction training may compete with reductions that were induced by acquisition (Lai et al., 2012). Synaptic plasticity is differentially modulated across different areas of the brain. Instead of fear conditioning resulting in the elimination of spines as observed by Lai et al. (2012), training correlates with an increase in both the size and number of spines in the anterior cingulate cortex (aCC) and the infralimbic (IL) portion of the PFC (Vetere et al., 2011). Extinction was again found to have opposing effects on the morphological changes brought about by fear conditioning, but they differed according to the region studied: the number of spines in the aCC decreased but they remained enlarged, whereas the number of spines in the IL-PFC remained constant, but diminished in size (Vetere et al., 2011). These examples illustrate that extinction training is able to reverse morphological changes induced by acquisition and implies that, at least to some extent, extinction may mediate a partial erasure of the original memory trace. However, these findings may be restricted to regions of the brain that are critically involved in cognitive flexibility such as the PFC, as it has been shown that in the amygdala, networks originally associated with fear conditioning are left intact and merely silenced by extinction (Repa et al., 2001; Herry et al., 2008). Furthermore, the neuronal circuits activated in the amygdala during fear acquisition are distinct to those activated during fear extinction (Herry et al., 2008).

Finally, an elegant study recently demonstrated that a subset of amygdala neurons which fire during fear conditioning (and which subsequently also fire upon retrieval) are not activated following fear extinction, due to structural remodeling within inhibitory perisomatic synapses (Trouche et al., 2013). This illustrates that extinction activity directly influences the structure of neurons that code for the original memory. In summary, the evidence indicates that extinction training interacts with the original fear circuit (which is unsurprising given that an extinction memory without reference to the original fear memory is essentially meaningless), and that structurally, extinction appears to oppose acquisition. However, this interaction may only result in partial suppression of the original memory trace due to the regional specificity associated with fear and extinction.

## Receptor signaling mechanisms

A number of neurotransmitters and their cognate receptors are important for the reconsolidation and extinction of retrieved memories, and pharmacological manipulation of the glutamatergic NMDA and AMPA receptors (NMDAR and AMPAR, respectively) has revealed that both have crucial roles in these processes (Baker and Azorlosa, 1996; Suzuki et al., 2004; Winters and Bussey, 2005; Ben Mamou et al., 2006; Yamada et al., 2009; Nikitin and Solntseva, 2013). Systemic administration of NMDAR antagonists can prevent both the reconsolidation of the original memory and the consolidation of an extinction memory (Eisenberg et al., 2003; Pedreira and Maldonado, 2003; Suzuki et al., 2004; Lee et al., 2006; Flavell and Lee, 2013). Interestingly, reconsolidation and extinction mechanisms do not appear to occur at the same time, with one process being preferred over the other. This has been interpreted as a trace dominance effect, in that only the dominant trace will be impaired (Eisenberg et al., 2003). In terms of destabilization, this implies that despite a reactivation session being procedurally the same as a short extinction session, destabilization cannot occur at the beginning of session and simultaneously destabilize both the original CS-US while promoting the consolidation of a new extinction memory. This is illustrated by the observation that administration of NMDA receptor antagonists or protein synthesis inhibitors during extinction impairs the consolidation of extinction, but not the reconsolidation of the original trace, and by the fact that reconsolidation can be preferentially targeted by varying the duration of exposure to the CS (Eisenberg et al., 2003; Pedreira and Maldonado, 2003; Suzuki et al., 2004; Flavell and Lee, 2013).

On the surface, the role of NMDARs in fear extinction and reconsolidation appears to be relatively well understood with activation potentiating both processes (Walker et al., 2002; Lee et al., 2006, 2009) and inhibition leading to an impairment (Cox and Westbrook, 1994; Baker and Azorlosa, 1996; Lee et al., 2006); however, there have been conflicting reports. The NMDAR antagonist ifenprodil prevents anisomycin-induced amnesia when injected into the basolateral amygdala (BLA) before, but not after, retrieval, suggesting that NMDARs are crucial for destabilization but not reconsolidation (Ben Mamou et al., 2006). This is at odds, however, with the many reports of NMDAR antagonists, in particular MK-801, preventing reconsolidation (Przybyslawski and Sara, 1997; Pedreira et al., 2002; Lee et al., 2006; Brown et al., 2008; Lee and Everitt, 2008; Winters et al., 2009; Flavell and Lee, 2013). MK-801 is a non-selective NMDAR antagonist, whereas ifenprodil specifically targets the NR2B subunit of the NMDAR complex (Williams, 1993). NR2B-containing NMDARs have been shown to suppress CREB, promote long-term depression (LTD) and activate protein degradation pathways (Hardingham et al., 2002), while GluN2A-containing NMDARs promote CREB phosphorylation and long-term potentiation (LTP; Liu et al., 2004). Recently, a double dissociation for the roles of NMDAR subtypes has emerged, with NR2A-containing NMDARs being required for reconsolidation, whereas NR2B-containing NMDARs are required for their destabilization (Milton et al., 2013), perhaps explaining what were previously paradoxical results and illustrating that the destabilization step may be entirely separate to reconsolidation.

Pharmacological blockade of AMPA receptors (AMPARs) has been shown to impair both the consolidation and retrieval of memories (Liang et al., 1994; Bast et al., 2005; Winters and Bussey, 2005), as well as their extinction (Walker and Davis, 2002; Zushida et al., 2007; Yamada et al., 2009) so it is somewhat surprising that AMPAR antagonists have been reported to have no effect on either destabilization or reconsolidation of fear memories (Ben Mamou et al., 2006; Milton et al., 2013). As is the case with NMDARs, different sub-populations of AMPARs are likely to be important for different mechanisms. Calcium-permeable AMPARs (CP-AMPARs) generally lack the GluA2 subunit, are less stable at synapses and have been associated with LTD, while calcium-impermeable AMPARs (CI-AMPARs), which do contain the GluA2 subunit are more stable, have been associated with LTP and make up the majority of basal AMPA activity (Isaac et al., 2007). Phosphorylation of AMPARs regulates receptor trafficking (Blackstone et al., 1994; Esteban et al., 2003) and there is an increase in phosphorylation of the GluR1 subunit at serine 845 associated with memory retrieval (Monfils et al., 2009; Jarome et al., 2012). Memory consolidation is associated with the increased expression of CI-AMPARs at synaptic sites but memory retrieval results in an abrupt exchange of CI-AMPARs for CP-AMPARS (Clem and Huganir, 2010; Rao-Ruiz et al., 2011; Hong et al., 2013a). Over a period of hours, the CP-AMPARs are gradually replaced with CI-AMPARs, an event that correlates with the “reconsolidation window” (Clem and Huganir, 2010; Rao-Ruiz et al., 2011; Hong et al., 2013a). Finally, preventing the exchange of AMPARs blocks destabilization and protects from anisomycin-induced amnesia, as does blockade of the newly inserted CP-AMPARs (Hong et al., 2013a). Thus, glutamate receptor trafficking mechanisms are crucial for the determination of whether a memory will undergo reconsolidation or extinction.

In addition to glutamate receptors, the roles of several other neurotransmitters have been investigated. The endogenous cannabinoid receptor, CB1, has been shown to prevent reconsolidation by blocking destabilization, along with L-type voltage-gated calcium channels, as they are able to protect memories from the effects of protein synthesis inhibitors applied during reactivation (Suzuki et al., 2008). Enhancing cannabinoid activity potentiates fear extinction (Marsicano et al., 2002; Chhatwal et al., 2005; Pamplona et al., 2006; de Oliveira Alvares et al., 2008), so it is possible that destabilization could also be required for extinction. A recent study has shown that CB1Rs were increased in neurons that are activated by both fear conditioning and subsequent fear extinction. This was thought to represent an attempt to preserve the original CS-US trace through the ability of CB1R activity to prevent the release of the inhibitory neurotransmitter γ-aminobutyric acid (GABA; Trouche et al., 2013). However, this seems to be at odds with its established role in the potentiation of fear extinction (Marsicano et al., 2002; Pamplona et al., 2006), so it is possible that it is having a different effect via modulation of protein-kinase and phosphatase pathways during extinction (Cannich et al., 2004) perhaps through a destabilization mechanism.

Given their established roles in varied cognitive functions linked with memory, it is very likely that the receptors for catecholamines and/or acetylcholine act as upstream triggers for the molecular mechanisms subserving memory destabilization. Reichelt et al. (2013) recently demonstrated the important role that dopaminergic transmission appears to play in appetitive memory destabilization. Dopaminergic activity mediated by the ventral tegmental area (VTA) is an important component of prediction error signaling in an appetitive Pavlovian goal-tracking task in rats; unexpected changes in the nature of reward are associated with a negative prediction error signal, which depends on VTA dopaminergic tone (Takahashi et al., 2009). Reasoning that prediction error, indicative of a potential memory updating situation, may be necessary for memory destabilization in such tasks, Reichelt et al. (2013) assessed the effects of VTA dopamine dysregulation on the memory reconsolidation process. Manipulation of VTA dopaminergic signaling, achieved via microinfusions of either the GABAergic agonists baclofen and muscimol or the D2 receptor antagonist sulpiride, prevented destabilization of the appetitive goal-tracking memory as evidenced by the failure of post-retrieval systemic injections of the NMDAR antagonist MK-801 to disrupt subsequent goal tracking. A follow-up experiment suggested that the VTA is not the site of memory storage for this task; rather, the dopaminergic signal from the VTA likely regulates destabilization of the memory in the nucleus accumbens or amygdala (Reichelt et al., 2013). Whether dopamine plays a similar role in destabilization of aversively motivated memories remains a question for future research.

Winters and colleagues have investigated the involvement of acetylcholine in object memory destabilization. In a previous study using the spontaneous object recognition paradigm for rats, the boundary conditions of memory age and encoding strength were shown to influence the likelihood of object memories becoming labile upon reactivation (Winters et al., 2009). Specifically, memories that were more strongly encoded at the time of initial learning or relatively more remote at the time of memory reactivation did not readily destabilize, such that post-reactivation MK-801 failed to disrupt object memory reconsolidation. However, when similar object memories were reactivated in the presence of an explicit novel cue—a salient floor insert with a novel texture placed in the testing apparatus during the reactivation phase—the memories were destabilized, and systemic post-reactivation MK-801 disrupted object recognition performance when assessed in a test phase 24 h later. This finding highlights the importance of novel information in rendering consolidated memories labile upon reactivation and is consistent with an updating role for the reconsolidation process.

Building on this interpretation, cholinergic transmission could contribute to this novelty-induced memory destabilization process, given the established roles for acetylcholine in various cognitive functions linked with new learning, such as attention, arousal, and novel memory encoding (Furey et al., 2000; Sarter et al., 2003; Hasselmo, 2006; Winters et al., 2006, 2007). Systemic administration of the muscarinic ACh receptor (mAChR) antagonist scopolamine blocks the reconsolidation impairment typically caused by systemic post-reactivation MK-801 when object memories are reactivated in the presence of a novel floor texture, thereby directly implicating mAChRs in the novelty-induced reactivation of strongly encoded and relatively remote object memories. Moreover, a highly similar result is seen when scopolamine is administered into the perirhinal cortex (PRh), a brain region commonly implicated in mammalian object recognition (Winters et al., 2011). Intra-PRh scopolamine administered before the reactivation phase appears to block memory destabilization, as it prevents the object memory reconsolidation deficit that is otherwise observed when intra-PRh anisomycin is infused immediately following the reactivation phase. Finally, enhancing cholinergic transmission with the mAChR agonist oxotremorine appears to mimic the memory destabilizing effects of novel information during reactivation. When object memories are strongly encoded or relatively remote, systemic co-administration of MK-801 and oxotremorine prior to reactivation leads to a significant impairment in reconsolidation. As noted above, MK-801 does not normally disrupt reconsolidation of such object memories under these conditions, and additional experiments also indicated that oxotremorine alone does not affect reconsolidation using these parameters. Thus, activating mAChRs with an exogenously administered drug appears to trigger the same cellular signaling cascade prompted by the presence of a salient novel cue in the reactivation phase, resulting in memory destabilization.

The exact nature of the intracellular mechanisms underlying the role of mAChRs in memory destabilization remains uncertain. However, mAChRs can influence both NMDAR- and AMPAR-mediated glutamatergic signaling (Segal and Auerbach, 1997; Fernandez de Sevilla et al., 2008; Fernandez de Sevilla and Buno, 2010). Indeed, activation of M1 mAChRs can produce post-synaptic insertion of AMPARs (Fernandez de Sevilla et al., 2008), which may provide a mechanistic link between the effects of cholinergic transmission on object memory destabilization and the previously reported requirement of AMPAR exchange for destabilization of fear memories (Hong et al., 2013b). Moreover, the effects of mAChR activation on AMPAR and NMDAR function are related to mAChR-induced stimulation of the inositol 1,4,5-triphosphate (IP_3_) second messenger cascade and may partly rely on activation of CaMKII activity (Fernandez de Sevilla et al., 2008; Fernandez de Sevilla and Buno, 2010). These findings suggest a potential connection between the effects of mAChR activation on object memory destabilization and the previously reported reliance of fear and object-in-place memory destabilization processes on protein degradation (Lee et al., 2008a; Choi et al., 2010). Like NMDAR, mAChRs may recruit the UPS via CaMKII activation (Bingol et al., 2010). There is a demonstrable link between M1 receptor activation and UPS-mediated protein degradation (Jiang et al., 2012); in this study, *in vitro* M1 receptor overexpression enhanced the degradation of β-site amyloid precursor protein cleaving protein 1 (BACE1), a protein that is elevated in sporadic Alzheimer's disease. Importantly, the muscarinic effect on BACE1 degradation was blocked by the proteasome inhibitor β-lac, suggesting that M1 receptors regulate BACE1 degradation via the UPS pathway (Jiang et al., 2012).

The bidirectional effects of cholinergic manipulations on object memory destabilization provide particularly strong evidence for a key role of acetylcholine transmission in this process. It will be important for future studies to assess whether these effects can be generalized to other forms of memory and in other brain regions known to demonstrate reconsolidation effects. A reliable role for acetylcholine in memory destabilization would have important implications for understanding age- and disease-related deficits in cognitive flexibility and could influence new thinking about remediation strategies for such conditions, as well as cases characterized by pervasive maladaptive memories, such as PTSD and phobias.

## Protein degradation

*De novo* protein synthesis is required for both memory consolidation (e.g., Flexner et al., 1963; Bourtchouladze et al., 1998; Hernandez et al., 2002; Bekinschtein et al., 2007; Duvarci et al., 2008; reviewed in Hernandez and Abel, 2008) and reconsolidation (e.g., Nader et al., 2000; Debiec et al., 2002; Morris et al., 2006; Duvarci et al., 2008; for a review see Alberini et al., 2006). However, a growing number of studies indicate that protein degradation also plays a key role in memory (e.g., Merlo and Romano, 2007; Artinian et al., 2008; Lee, 2008, 2010; Lee et al., 2008b, 2012; for reviews see Kaang et al., 2009; Fioravante and Byrne, 2011; Kaang and Choi, 2012; Jarome and Helmstetter, 2013). Protein degradation is mediated, in large part, by the ubiquitin-proteasome system (UPS), in which proteins are marked for degradation by polyubiquitination (Nandi et al., 2006). This process occurs in almost every mammalian cell; however, within the brain it can be modulated by neuronal activity. Depolarization of cultured hippocampal neurons leads to a rapid redistribution of the proteasome complex into dendritic spines (Bingol and Schuman, 2006), and changes in synaptic activity can increase the ubiquitination and turnover of plasticity-related proteins within the post-synaptic density (Ehlers, 2003). UPS activity is modulated by calcium ion entry via N-methyl-D-aspartate receptors (NMDARs) and by L-type voltage gated calcium channels (LVGCCs), which in turn activate calcium/calmodulin-dependent protein kinase II or CaMKII (Djakovic et al., 2009). Furthermore, impairment of UPS activity affects long-term potentiation (Fonseca et al., 2006; Karpova et al., 2006; Dong et al., 2008; Cai et al., 2010; Pick et al., 2013), indicating that it has a key role in synaptic plasticity.

There are several reports of UPS blockade leading to deficits in learning and memory, fear conditioning leads to an increase in polyubiquitination in the amygdala, and infusion of clasto-lactacystin-beta-lactone (β-lac), a proteasome inhibitor, immediately after training, results in a deficit in both contextual and cued fear (Jarome et al., 2011). However, this is somewhat controversial as others have observed no effect on contextual fear following the disruption of protein degradation (Lee, 2008, 2010; Lee et al., 2008b; Pick et al., 2013; Ren et al., 2013). There is evidence that UPS disruption impairs the consolidation of spatial memory tasks (Lopez-Salon et al., 2001; Merlo and Romano, 2007; Artinian et al., 2008), which may indicate that spatial but not emotional memories require protein degradation for consolidation.

While its role in memory consolidation is unclear, several studies have demonstrated that synaptic protein degradation is a critical step in the destabilization that occurs prior to reconsolidation (Lee, 2008, 2010; Lee et al., 2008b, 2012; Jarome et al., 2011; Ren et al., 2013). Following the reactivation of a contextual fear memory, an increase in polyubiquitination can be observed, in a selective manner, within the hippocampus (Lee et al., 2008b). The ubiquitination of proteins leads to their degradation, and this decrease can be blocked with β-lac (Lee et al., 2008b), indicating that the reactivation of previously acquired memories leads to a specific pattern of protein degradation at the synapse.

Inhibition of protein synthesis prevents reconsolidation and leads to profound amnesia (Nader et al., 2000; Debiec et al., 2002; Morris et al., 2006); however, infusion of a proteasome inhibitor in conjunction with the protein synthesis inhibitor prevents reactivation-dependent amnesia (Lee, 2008, 2010; Lee et al., 2008b; Jarome et al., 2011; Ren et al., 2013). Infusion of a proteasome inhibitor alone during reactivationl has no effect on the original memory (Lee, 2008, 2010; Lee et al., 2008b; Jarome et al., 2011; Ren et al., 2013; but see Artinian et al., 2008 who do report an effect), suggesting that protein degradation is required for destabilization of the reactivated memory, while protein synthesis is required for its reconsolidation (re-stabilization). Furthermore, it implies that reconsolidation cannot occur without first destabilizing the original memory. Protein degradation has been shown to be important for the strengthening of previously acquired contextual fear memories (Lee, 2008). An infusion of β-lac prior to a second training session prevented further learning observed in vehicle-treated animals, indicating that inhibition of protein degradation blocked strengthening, while leaving the original memory intact (Lee, 2008). Similarly, inhibiting protein synthesis blocks memory updating, as β-lac infusion after foot shock prevented the association of this aversive stimulus with a previously neutral context (Lee, 2010).

Preventing the destabilization of a previously acquired memory with a proteasome inhibitor has been reported in invertebrates at both a behavioral and a cellular level (Lee et al., 2012) and in addition to the aversive paradigms discussed above, has also been observed during the recall of appetitive (Ren et al., 2013) and spatial memories (Da Silva et al., 2013) in vertebrates. The conclusion that protein degradation is a crucial step in the destabilization of memories before reconsolidation is supported by the fact that blockade of LVGCCs (known to be upstream activators of the UPS) will also block anisomycin-mediated amnesia at retrieval, while leaving the original memory intact (Suzuki et al., 2008).

Finally, there is emerging evidence that preventing synaptic protein degradation can disrupt extinction learning (Lee et al., 2008b; Mao et al., 2008; Pick et al., 2013; Ren et al., 2013). Proteasome inhibitors impair extinction (Lee et al., 2008b; Ren et al., 2013) and block D-cycloserine-mediated enhancement of extinction (Mao et al., 2008). Moreover, a Cdh1 (a subunit of ubiquitin E3 ligase and a crucial enzyme in the UPS) knock-out mouse exhibits profound extinction deficits (Pick et al., 2013). In summary, the current evidence indicates that protein degradation is required by both reconsolidation and extinction mechanisms, and it has been proposed that degradation is a key component of destabilization. Some authors have gone on further to suggest that the requirement of protein degradation during extinction represents a destabilization of the original fear memory allowing a partial erasure or “unlearning” (Lee et al., 2008b). This explanation seems unlikely, because as was described above, if the original CS-US memory is destabilized during extinction then protein synthesis inhibitors would block reconsolidation of the original memory rather than disrupting extinction. Given that protein degradation does not appear to be a pre-requisite for the consolidation of new fear memories (Lee, 2008, 2010; Lee et al., 2008b; Pick et al., 2013; Ren et al., 2013), it is possible that it is specific to the process of extinction and could represent an interaction with the original memory through a mechanism other than reconsolidation.

## Epigenetic mechanisms and gene regulation

Dynamic changes in chromatin structure play a vital role in altering gene expression and are necessary for memory formation (Graff and Tsai, 2013). For example, distinct patterns of histone acetylation at the site of the brain-derived neurotrophic factor (BDNF) gene have been associated with both the acquisition of fear and its extinction. (Bredy et al., 2007). Histone modifications also play a role in the reconsolidation of a fear memory, as reconsolidation is associated with an increase in histone 3 (H3) acetylation (Maddox and Schafe, 2011) and blockade of histone deacetylation prevents the reconsolidation of strong memories (Federman et al., 2012). As such, it has been suggested that histone modification and the enzymes that mediate these changes in chromatin state may contribute to memory reconsolidation by destabilizing fear memory (Bredy and Barad, 2008; Maddox et al., 2013a,b). For example, the histone acetlytransferase (HAT) p300/CBP-associated factor (PCAF) has also recently been implicated in memory formation. PCAF knock-out mice exhibit impaired spatial learning and difficulty in adapting to reversal of an operant conditioning task (Maurice et al., 2008). Nuclear expression of PCAF within the IL-PFC is increased following extinction training and there is evidence that PCAF is vital for LTP within this region (Wei et al., 2012). Following administration of the PCAF activator SPV106, a marked reduction in renewal of conditioned fear has been observed (Wei et al., 2012). Thus, this finding supports the role of PCAF in promoting the formation of extinction memory.

MicroRNAs (miRNAs) are a distinct class of small non-coding RNAs, which have important roles in epigenetic regulatory mechanisms. They belong to a family of endogenously expressed small regulatory RNAs that post-transcriptionally regulate gene silencing in plants, invertebrates, and mammals by inhibiting the function of their target mRNAs through complementary binding (Bartel, 2004). A unique feature of these non-coding RNAs is their ability to bind and regulate many genes, and in some cases multiple miRNAs target similar families of genes (Krichevsky et al., 2003; John et al., 2004; Lim et al., 2005; Friedman et al., 2009; Hendrickson et al., 2009), thereby enhancing their ability to regulate plasticity in the brain. The transient nature of miRNAs, their localized expression in dendrites, their capacity to respond in an activity-dependent manner, and the fact that a single miRNA can simultaneously regulate many genes, make brain-specific miRNAs, together with other non-coding regulatory RNAs, ideal candidates for the fine-tuning of gene expression associated with neural plasticity and memory formation.

Across three different learning paradigms, Nudelman et al. (2010) found that hippocampal expression of miR-132 consistently peaks 45 min after training and returns to baseline within 90 min, perhaps indicating a general role for miR-132 in learning processes such as encoding or the initial phase of memory consolidation. Similarly, we have recently observed that another brain-specific miRNA, miR-128b, is induced within the PFC 2 h after fear extinction training but returns to baseline within 6 h of training (Lin et al., 2011). This transient, learning-induced increase in miRNA expression in the adult brain bears a striking resemblance to the pattern of expression typically reserved for plasticity-related immediate early genes such as c-fos, Arc or zif268, further suggesting a regulatory function for miRNA activity in learning and memory. There are regional and cell type-specific miRNAs that participate in the regulation of gene function in a learning-dependent manner. In the case of miR-128b, our early evidence indicates that this miRNA, expressed within neurons innervated by dopamine in the PFC, may be intimately related to the formation of fear extinction memory. Given that the initial phase of fear extinction learning involves competition with a previously acquired fear memory trace, this transient increase in miR-128b expression may serve to temporarily inhibit the expression of plasticity-related genes associated with retrieval of the original fear, in order to allow the formation of fear extinction memory to proceed.

It has been reported that miR-128b targets a protein called regulator of calmodulin (RCS), which is a competitive inhibitor of the protein phosphatase calcineurin (CaN) (Rakhilin et al., 2004). CaN has been shown to regulate the strength of aversive memory (Baumgartel et al., 2008) and an increase in CaN activity is essential for the formation of extinction memory (Lin et al., 2003; Havekes et al., 2008). Recent evidence also suggests that this protein phosphatase may exert its effect on fear extinction memory by destabilizing the original fear-related memory at the time of retrieval (de la Fuente et al., 2011; Shaw et al., 2012). Thus, given the labile state of the fear memory during extinction and inhibition of the reconsolidation by CaN, the evidence suggests that there is at least some capacity for degradation of the strength of the original fear memory, which can be mediated indirectly by miR-128b through increased CaN activity at the time of retrieval and lead to enhanced extinction (Lin et al., 2011).

## Outlook

It is evident that the relationship between reconsolidation and extinction is intimately related to cellular and molecular mechanisms engaged at the time of retrieval. Factors influencing this process include structural modifications to dendrites, the stability of synaptic proteins, membrane-bound receptor signaling, intracellular signal transduction and dynamic changes in chromatin states within the nucleus that are mediated by epigenetic regulatory mechanisms. There is evidence that promotion of reconsolidation or extinction is dependent on the activity of the transcription factor nuclear factor kappa B (NF-κ B). Reactivation of fear memories leads to a number of protein phosphorylation cascades, including the ERK-MAPK (extracellular signal-regulated kinase—mitogen-activated protein kinase) pathway (Duvarci et al., 2005) and the IKK (inhibitor kappa B kinase) pathway (Lubin and Sweatt, 2007). Recently, it has been demonstrated that reconsolidation is specifically associated with the activation of the IKK/NF-κ B pathway (Lee and Hynds, 2013) and a consequent increase in NF-κ B (Merlo et al., 2005), while extinction is associated with an active suppression of NF-κ B (Merlo and Romano, 2008). These studies provide a convincing explanation as to how procedurally similar experiences can result in a markedly different outcome.

Since it is presumed that the parameters of the reactivation process, in particular duration or frequency of exposures to the CS are critical in determining whether reconsolidation or extinction will occur (Pedreira and Maldonado, 2003), it seems that the reactivation of a memory describes a process in which initially, protein phosphorylation pathways leads to the levels of transcription factors such as NF-κ B to initially rise, thus promoting reconsolidation, but the prolonged presentation of the CS (extinction) will lead to an inhibition of reconsolidation apparatus, thereby promoting extinction. This requirement for transcription factor activity to be altered over the course of the reactivation/extinction session further implies that destabilization is distinct to the process of reactivation and that destabilization may occur at the end of the session, once the reconsolidation or extinction pathway has been determined. This would suggest that reconsolidation and extinction cannot occur at the same time and, therefore, that extinction does not involve a concurrent suppression of the original fear memory through reconsolidation. It does not, however, exclude the possibility that the original memory is being suppressed through another mechanism that does not require protein synthesis.

Future studies should also consider other factors that influencing reactivation, which are likely to be important for memory destabilization and extinction. These include electrical signaling mediated by voltage-gated calcium channels (Suzuki et al., 2008) and gap junctions (Bissiere et al., 2011), and other classes of non-coding RNAs. The expansion of transcriptionally active long non-coding sequences (lncRNA) in the mammalian genome, in particular, appears to have occurred primarily in species with higher-order cognitive function (McLean et al., 2011), and brain-enriched lncRNAs are expressed in both a spatiotemporal- and cell-type-specific manner (Mercer et al., 2008). Conservative annotations estimate that there are at least 9500 independent lncRNA genes, several of which have been implicated in neocortical development (Bond and Fox, 2009), neurogenesis (Ng et al., 2012), and synaptogenesis (Bernard et al., 2010). Importantly, these enigmatic non-coding RNAs function as decoys for transcription-related factors, as modular scaffolds or as guides to direct chromatin-modifying complexes to their genomic sites of action (Rinn and Chang, 2012; Spadaro and Bredy, 2012; Mercer and Mattick, 2013). Thus, given their rapid rate of turnover, brain-specific lncRNAs are uniquely positioned to mediate rapid genomic responses to external stimuli in a manner distinct from, and more complex than, the much slower acting protein-coding genes (Clark et al., 2012), they are therefore likely to be involved in the rapid cellular and molecular responses required for memory destabilization at the time of reactivation leading to either reconsolidation or extinction of various forms of memory.

### Conflict of interest statement

The authors declare that the research was conducted in the absence of any commercial or financial relationships that could be construed as a potential conflict of interest.
